# Sigmoid isostiffness-lines: An *in-vitro* model for the assessment of aortic stenosis severity

**DOI:** 10.3389/fcvm.2022.960170

**Published:** 2022-10-05

**Authors:** Eric Buffle, Michael Stucki, Shaokai Zheng, Maxime Chiarelli, Christian Seiler, Dominik Obrist, Stefano F. de Marchi

**Affiliations:** ^1^Department of Cardiology, University Hospital Bern, Bern, Switzerland; ^2^ARTORG Center, University of Bern, Bern, Switzerland

**Keywords:** aortic stenosis, isostiffness lines, valvular heart disease, low-flow, low-gradient aortic stenosis, echocardiography, *in-vitro* model, machine learning

## Abstract

**Introduction:**

The aortic valve opening area (AVA), used to quantify aortic stenosis severity, depends on the transvalvular flow rate (Q). The currently accepted clinical echocardiographic method assumes a linear relation between AVA and Q. We studied whether a sigmoid model better describes this relation and determined “isostiffness-lines” across a wide flow spectrum, thus allowing building a nomogram for the non-invasive estimation of valve stiffness.

**Methods:**

Both AVA and instantaneous Q (Q_inst_) were measured at 10 different mean cardiac outputs of porcine aortic valves mounted in a pulsatile flow loop. The valves' cusps were chemically stiffened to obtain three stiffness grades and the procedure was repeated for each grade. The relative stiffness was defined as the ratio between LV work at grade with the added stiffness and at native stiffness grade. $\bar{{\text{AVA}}_{\text{peak}}}$ corresponding to the selected $\bar{{\text{Q}}_{\text{peak}}}$ of the highest 3 and 5 cardiac output values was predicted in K-fold cross-validation using sequentially a linear and a sigmoid model. The accuracy of each model was assessed with the Akaike information criterion (AIC).

**Results:**

The sigmoid model predicted more accurately $\bar{{\text{AVA}}_{\text{peak}}}$ (AIC for prediction of AVA with $\bar{{\text{Q}}_{\text{peak}}}$ of the 3 highest cardiac output values: –1,743 vs. –1,048; 5 highest cardiac output values: –1,471 vs. –878) than the linear model.

**Conclusion:**

This study suggests that the relation between AVA and Q can be better described by a sigmoid than a linear model. This construction of “isostiffness-lines” may be a useful method for the assessment of aortic stenosis in clinical echocardiography.

## 1. Introduction

Patients with symptomatic severe aortic stenosis, one of the two most common valvular heart diseases, benefit from aortic valve replacement (1). This underlines the importance of a correct diagnosis. The aortic valve opening area (AVA) is the main parameter used to assess the severity of aortic stenosis (1). However, AVA depends on the transvalvular flow rate (Q) and the nature of this relation is unclear. Around a third of patients with severe aortic stenosis have reduced Q due to reduced left ventricular function (2, 3). This situation is ambiguous because the reduced AVA can be due to reduced Q alone, with or without increased stiffness of the valve. However, aortic valve replacement is indicated only for stiffened valves (e.g., due to calcification). Dobutamine stress echocardiography is used as an additional test to increase Q and observe the corresponding change in AVA (4–6). The main assumption, currently used for clinical decisions, is that the relation between Q and AVA is linear. Previous study showed that the relation between Q and transvalvular pressure loss (ΔP) under stress is non-linear and difficult to predict (7) and that severe aortic stenosis does not seem to behave like an orifice with a fixed area (8) To account for the large interindividual variability of Q-increases during dobutamine stress, the AVA has been projected to a standardized Q-value (set arbitrarily to 250 ml/s) using linear interpolation (3). The corollary of the assumption of linearity, however, is that AVA would always continue to increase without boundaries with increasing Q. In this *in vitro* experiment with varying stiffness grades of porcine aortic valves, we compared the accuracy of a linear and a sigmoid, saturating model for the prediction of valve stiffness and AVA. We constructed “isostiffness-lines” over a large spectrum of Q that also include values encountered during low-flow situations and stress tests.

## 2. Methods

**Figure 1:**
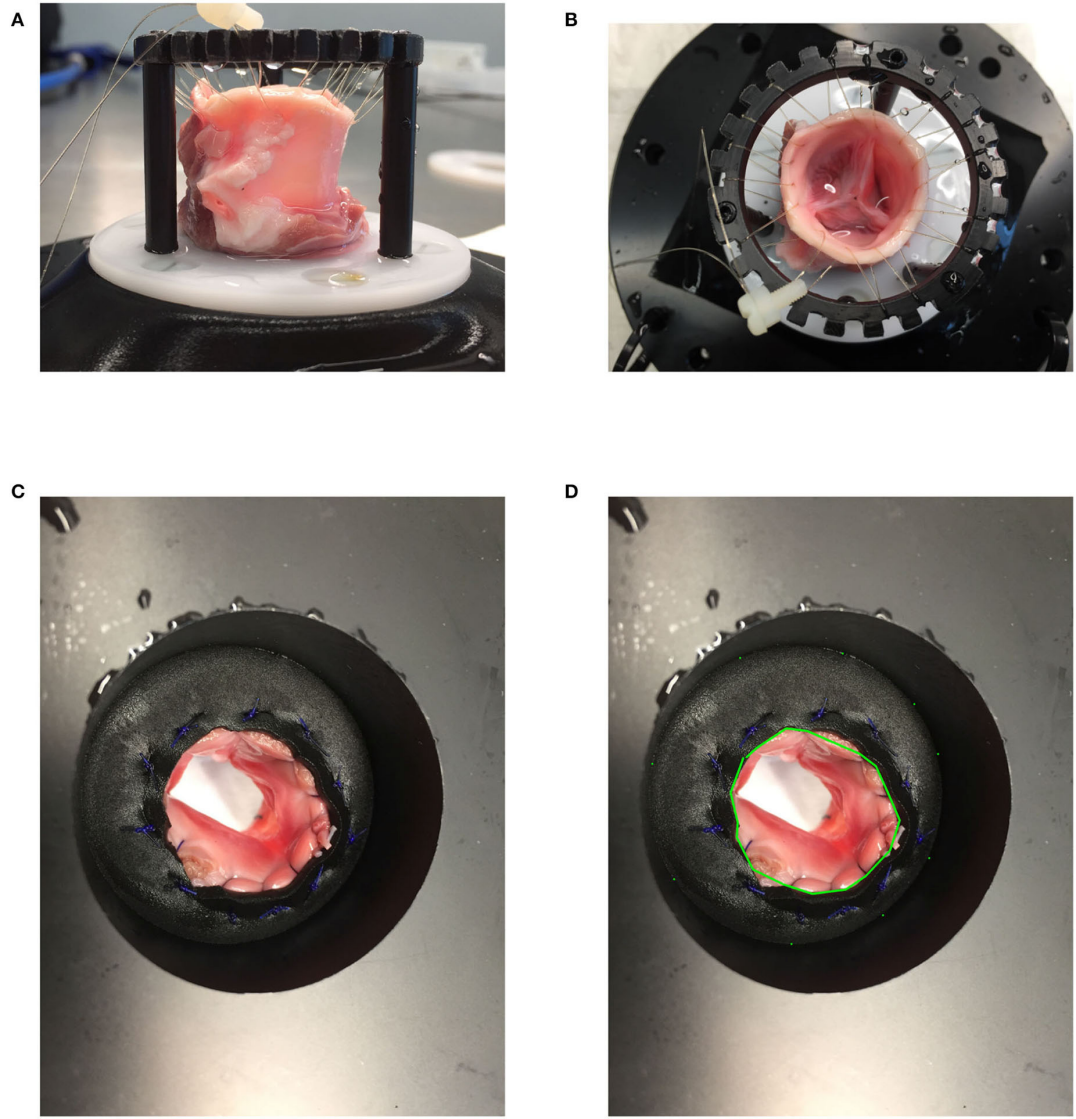
**(A,B)** Valve mounting: A harvested valve loosely attached to a ring with a sewing thread on the side of the ascending aorta and sewn to a neoprene sheet entrapped between two POM (Polyoxymethylene) flanges. **(C,D)** LVOT area measurement.

**Figure 2:**
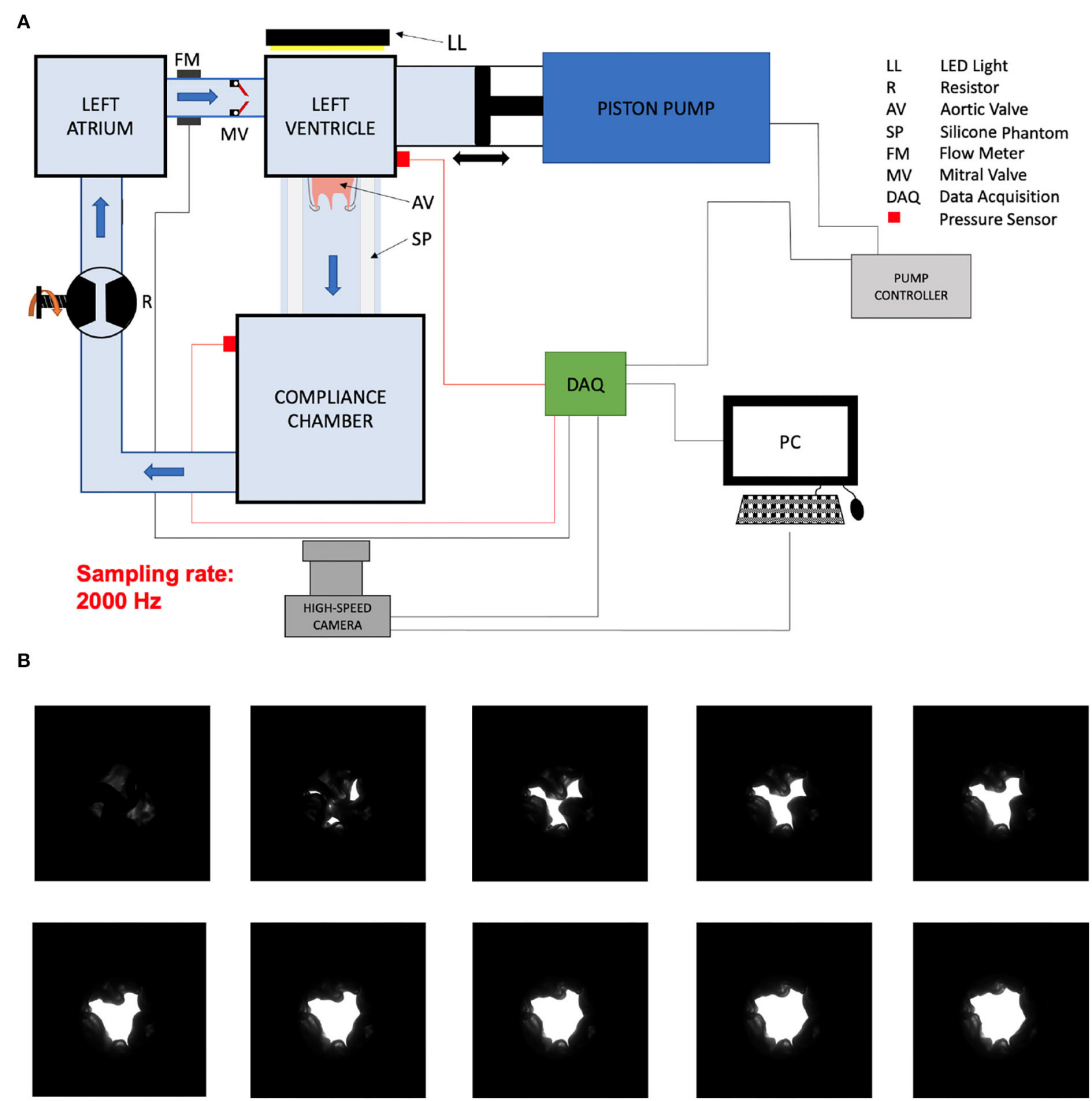
Sketch of the setup **(A)**. Video frames of valve opening **(B)**.

We harvested aortic valves from 4 months old pigs (≈ 120 kg) which were slaughtered within 24 h and kept thereafter at 4°C before the preparation of the valves. A valve identifier scheme was defined as follows: AXXX, where A stands for aortic valve and XXX is the ID number of the valve starting from 001 defined as the harvested valve number. We cut the valve with human surgical instruments as follows: on the side of the left ventricle (LV), we preserved 1 cm of the left ventricular outflow tract (LVOT) below the lower plane defined by the cusps of the aortic valve and cut the ascending aorta 0.5 cm above the plane defined by the 3 commissures of the aortic valve. We then sutured the LVOT on a wedge of neoprene sheet with a central hole. We then secured the neoprene sheet with the sutured valve between two POM (Polyoxymethylene) flanges clamped together with screws (Figures 1A,B). We sutured the aortic side of the valve to a loosely tied indented ring so that the valve cusp would not collapse during diastole, thus allowing the proximal ascending aorta to dilate during systole. We measured the area of the LVOT by counting the number of pixels within the LVOT in an image of the mounted valve taken with the camera in the axial direction from the ventricle side. We calibrated the pixel size by measuring the number of pixels of the inner portion of a circular hole of the known area of the flange on the same image (Figures 1C,D). We placed the valve inside a distal ascending aorta phantom made of silicone (ELASTOSIL^®^RT 601 A/B Wacker Chemie AG, München, Germany). The aortic valves were tested in a flow loop simulating the left heart as described previously (9). The cardiac output was measured by a transit-time flow probe (TS410/ME-11PXL, Transonic Systems, Inc., Ithaca, NY, USA) which was positioned directly upstream of the mechanical mitral valve between the left atrium and the LV (Figure 2A). The blood mimicking fluid, composed of 40/60% (by weight) glycerine and deionized water at room temperature was used to mimic the viscosity of the blood (9). We recorded the pressure with pressure transducers in the LV (XtransVR, CODAN pvb Critical Care GmbH, Forstinning, Germany) and in the compliance chamber (PBMN flush, Baumer Electric AG, Switzerland) of the flow loop. The two pressure sensors were calibrated with a water column. The distance between the two pressure sensors was 23.2 cm and the distance between the valve and the pressure sensor was 20.5 cm (with a length of the ascending aorta phantom of 15.5 cm, the pressure sensor residing 5 cm inside the compliance chamber. The signals of the pump position, flow-meter, pressure in the LV and the compliance chamber, and the trigger were acquired *via* a data acquisition system (DAQ USB-6221, National Instruments, Austin, Texas, USA) at a sampling frequency of 20,000Hz.

### 2.1. Aortic valve opening area (AVA)

Aortic valve opening area of the mounted aortic valves in a pulsatile flow loop was filmed during the ejection time with a high speed camera with a frame rate of 2,000 Hz (Photron FASTCAM Mini AX 100, Reutlingen, Germany).

A light source was placed behind the valve and the image contrast was optimized before acquisition. The image was binarized for every pixel during post-processing to dichotomize valve tissue and AVA (Figure 2B). The pixels were counted and the pixel size was measured by optically measuring a calibration checkerboard with squares of known size while keeping the same camera focus and focal length. The AVA divided by the LVOT area was reported for each valve in order to account for different valve sizes.

### 2.2. Transvalvular flow rate (Q)

**Figure 3:**
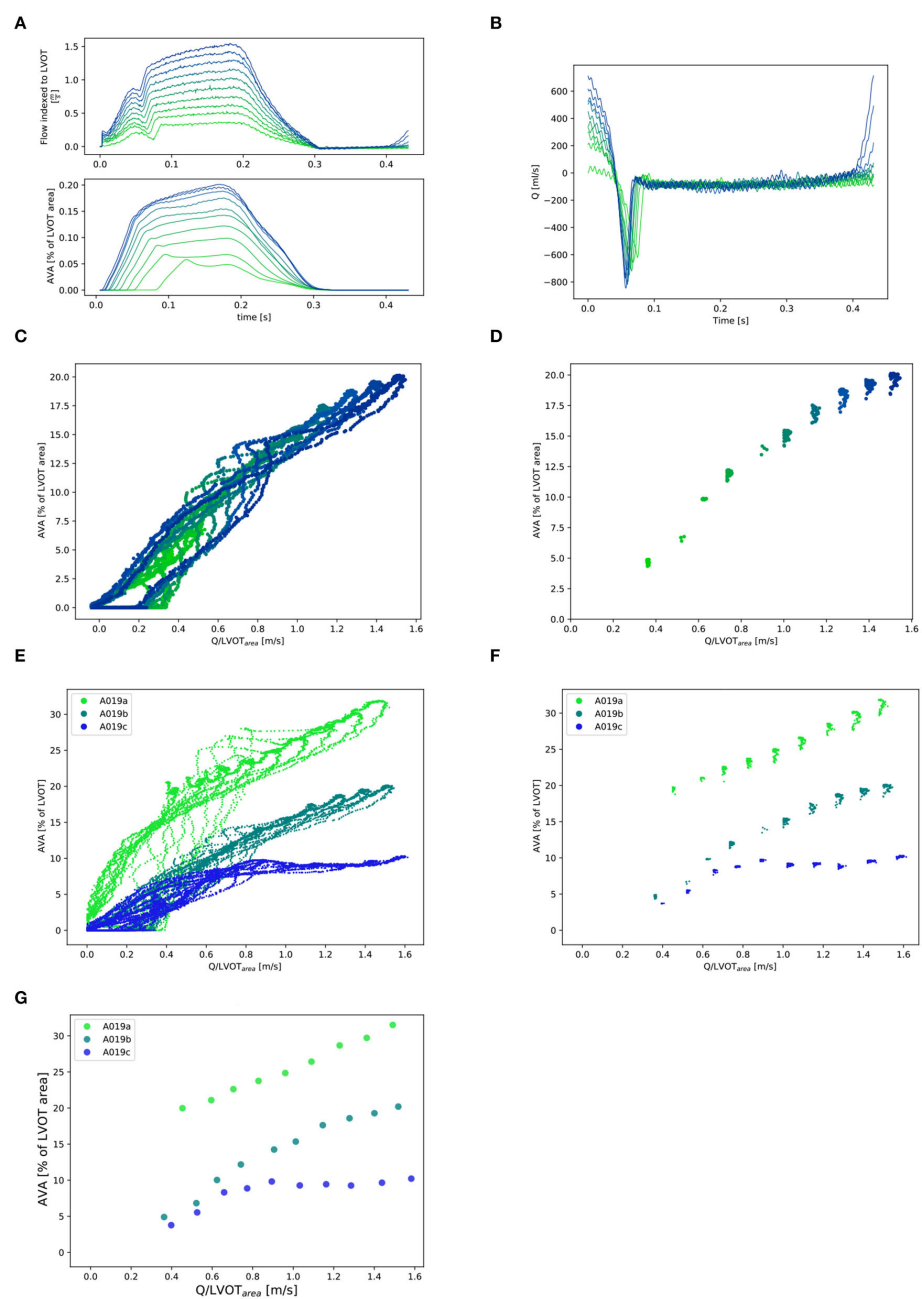
Selection of $\bar{{\text{Q}}_{\text{peak}}}$ and corresponding $\bar{{\text{AVA}}_{\text{peak}}}$ points (for one valve: A019). **(A)** Q_*inst*_ with synchronous corresponding AVA_*inst*_. Ten lines corresponding to the 10 different cardiac output values for one stiffness grade from 0.5l/min (in light green) to 5.0l/min (in dark blue). Stiffness grade 1 is depicted (cardiac output of 0.5l/min was missing at stiffness grade 0). **(B):** Instantaneous retrograde Q (Q _*retro*_) measured with flow probe placed proximal to the mitral valve for one stiffness grade (1). **(C)**: All Q_*inst*_ and corresponding AVA_*inst*_ for one valve at one stiffness grade 1 and 10 cardiac output values. **(D)**: Selection of the Q_*inst*_ higher than 97% of max{Q_*inst*_} and corresponding AVA_*inst*_ for one valve at one stiffness grade (1) and 10 cardiac output values. **(E)**: All Q_*inst*_ and corresponding AVA_*inst*_ for one valve at the three stiffness grades and 10 cardiac output values. **(F)**: Selection of the Q_*inst*_ higher than 97% of max{Q_*inst*_} and corresponding AVA_*inst*_ for one valve at three stiffness grades 1 and 10 cardiac output values and their mean ($\bar{{\text{Q}}_{\text{peak}}}$ and $\bar{{\text{AVA}}_{\text{peak}}}$) for each stiffness grade and each cardiac cycle: **(G)**.

**Figure 4:**
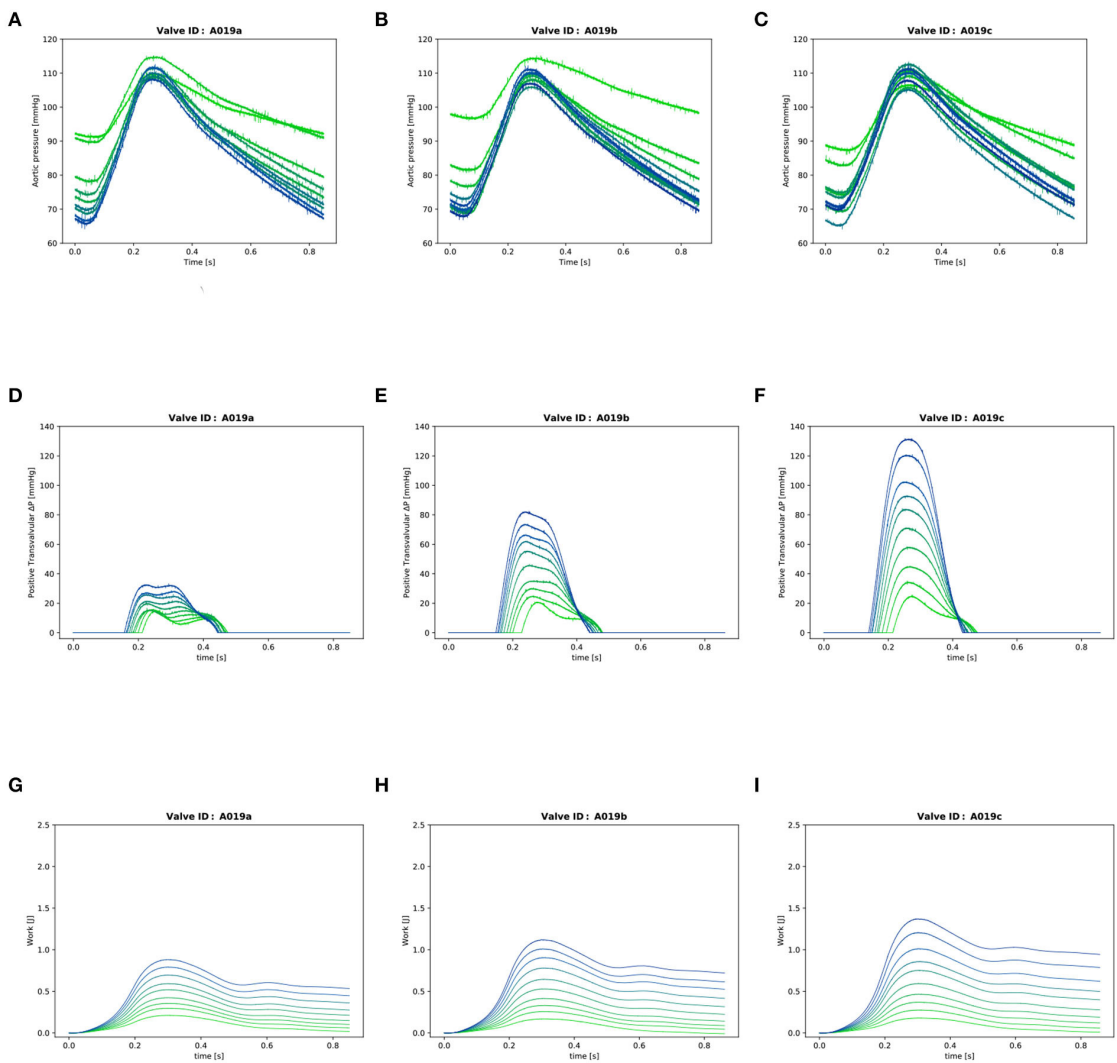
Valve A019: **(A–C)**: Instantaneous aortic pressure. **(D–F)**: Instantaneous transvalvular pressure. **(G–I)**: Instantaneous LV work. In each plot, there are 10 lines corresponding to the 10 different cardiac output values from 0.5l/min (in light green) to 5.0l/min (in dark blue) for one stiffness grade.

The instantaneous Q_inst_ was calculated from the piston velocity of the pump multiplied by the area of the piston. The retrograde flow (Q_retro_) measured by the flow sensor positioned proximal to the mechanical mitral valve was subtracted. This resulted in a notch in the flow signal (Figure 3A). In order to impose the same vascular afterload in all experiments, the resistor and the water level of the compliance chamber of the flow loop were adjusted to obtain a constant systolic pressure of 110 mmHg and a diastolic pressure of 70 mmHg (Figures 4A–C). The mean systolic transvalvular flow ($\bar{{\text{Q}}_{\text{syst}}}$) was computed by taking the average of all the Q_inst_ values over the ejection time. Both $\bar{{\text{Q}}_{\text{syst}}}$ in [ml/s] [as commonly used in the clinical literature (3, 4)] and $\bar{{\text{Q}}_{\text{syst}}}$ indexed to the LVOT area in [m/s] were reported in order to account for different valve sizes. For each time point, both AVA and Q_inst_ were measured at 10 different cardiac output values ranging from 0.5 to 5.0 liters/min (Figure 3).

### 2.3. Transvalvular gradient

The instantaneous transvalvular pressure gradient (ΔP) was computed by subtracting the pressure in the compliance chamber from the pressure in the LV (Figures 4D–F) which were recorded with the two transducers in LV and compliance chamber as described in Section 2. The mean transvalvular gradient was computed by averaging all the positive values during valve patency.

### 2.4. Cumulative LV work

The cumulative work performed by the LV was calculated for each time point of the cardiac cycle as previously described (10) (Figures 4G–I):


(1) $$\begin{array}{l} \begin{array}{l} W_{LV}(T)=\int_{0}^{T}P_{LV}\frac{dV_{LV}}{dt}\cdot dt \end{array} \end{array}$$

where *W*_*LV*_(*T*) is the cumulative work performed by the pump from the start of the cycle to the time point *T*, *P*_*LV*_ is the pressure in the LV and *dV*_*LV*_ is the instantaneous change in volume in the LV.

### 2.5. Valve stiffening and relative stiffness computation

The valves were stiffened by treating them with formaldehyde, a protein cross-linking agent, to obtain a total of three stiffness grades (stiffness grades *a, b*, and *c*). The relative stiffness s of the native stiffness grade a was defined as s_*a*_ = 1 and the relative stiffness of grades *b* and *c* was computed as ratio (*k*) between the LV work at grades *b* and *c* and the LV work at grade a at the four highest cardiac output values as follows:


(2) $$\begin{array}{l} \left[\begin{array}{c} W_{max\;5.01/min}^{a}\cdot k_{5.0}^{b,c}\\ W_{max\;4.51/min}^{a}\cdot k_{4.5}^{b,c}\\ W_{max\;4.01/min}^{a}\cdot k_{4.0}^{b,c}\\ W_{max\;3.51/min}^{a}\cdot k_{3.5}^{b,c} \end{array}\right]=\left[\begin{array}{c} W_{max\;5.01/min}^{b,c}\\ W_{max\;4.51/min}^{b,c}\\ W_{max\;4.01/min}^{b,c}\\ W_{max\;3.51/min}^{b,c} \end{array}\right] \end{array}$$

(3) $$\begin{array}{l} s^{b,c}=\frac{k_{5.0}^{b,c}+k_{4.5}^{b,c}+k_{4.0}^{b,c}+k_{3.5}^{b,c}}{4} \end{array}$$

where $W_{\text{max }}=\underset{0\leq T\leq T_{\text{cycle}}}{\max}\{W_{\text{LV}}(T)\}$ is the LV work or the work performed by the ventricle over the whole cycle at one particular stenosis grade and one particular cardiac output value. The average of the ratios of the four highest cardiac output values was calculated, corresponding to the range of physiological cardiac output values.

### 2.6. Post-processing

**Figure 5:**
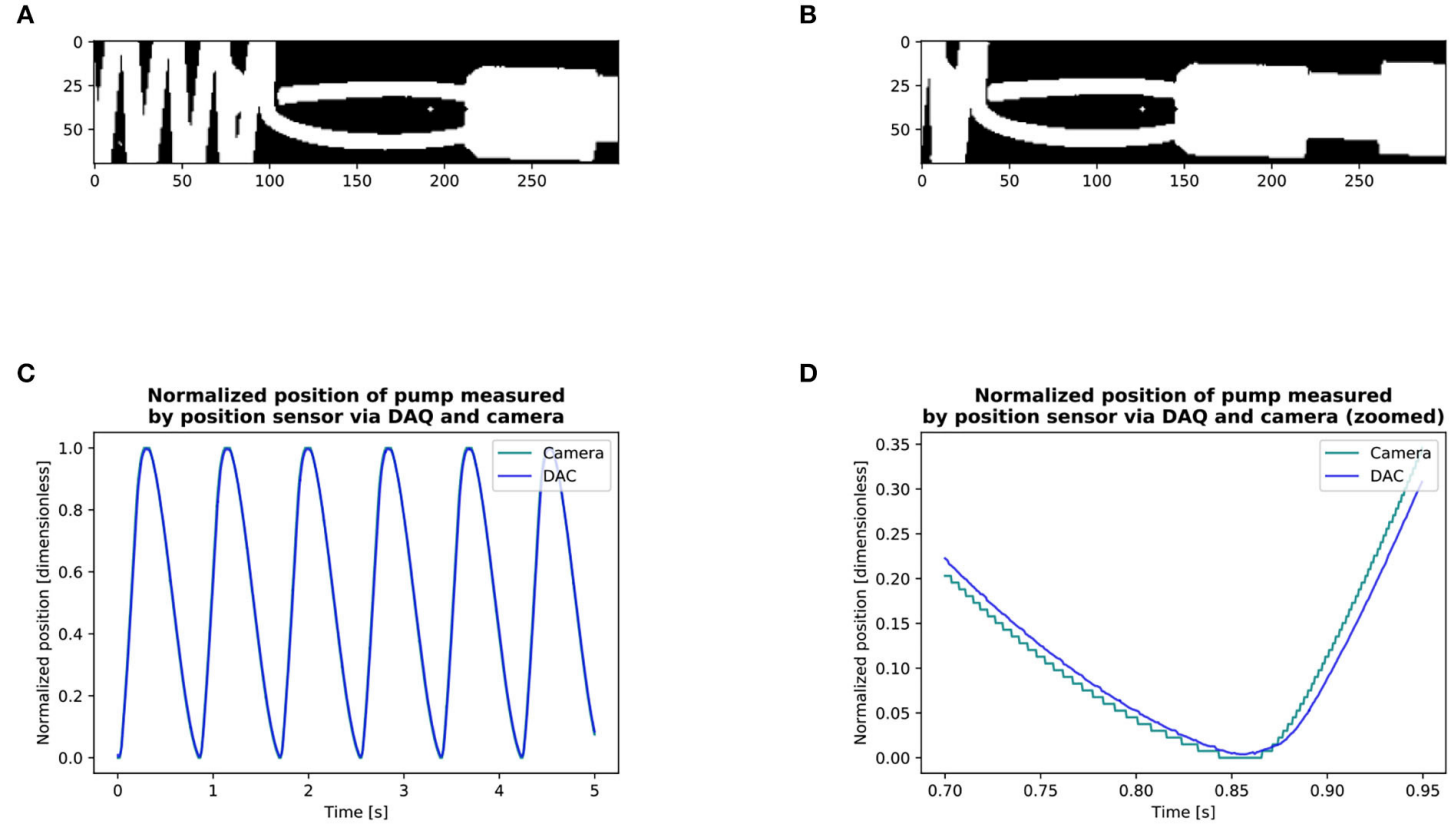
Measurement of delay between the camera and pump position sensor *via* the DAQ **(A,B)**: Detection of the position of the pump with the camera. **(C,D)**: Circular cross-correlation results of the position of pump: DAQ vs. camera signal. We obtained the signal of the position of the pump through the DAQ and filmed its displacement with the camera. We then made a binary copy of the region of interest and searched the leading edge of the pump by setting a point on a horizontal line in front of the pump on its displacement axis. For each frame, we searched the first white pixel, marked it as black for quality control, and thus recorded the pump position through the entire circle **(A,B)**. The entire series included 9 cycles with the camera and 9 with the position sensor *via* the DAQ. We then calculated the lag between the camera and the position of the pump *via* the DAQ using circular cross-correlation. We found that the signal of the camera was in advance of 14 ms with respect to the DAQ **(C,D)**. The circular cross-correlation of two signals *x, y*, ∈ size of *N* can be defined by [§8.8.1, Smith (11)]: ${\hat{r}}_{x,y}\left(l\right)=\frac{1}{N}\left(x⋆y\right)\left(l\right)=\frac{1}{N}\overset{N-1}{\underset{n=0}{\sum }}\bar{x\left(n\right)}y\left(n+l\right)$ *l* = {0, 1, 2, . . . , *N* − 1} Where ⋆ is the Discrete Fourier Transform correlation operator. The delay between the camera and the flow probe measured in another experiment was of 11 ms.

The delay between the camera and the pump position sensor was measured as well as the delay between the camera and the flow probe to synchronize the three signals using a circular cross-correlation [Figure 5; (11)]. The AVA signal was smoothened by performing a centered moving average over 40 frames (0.02 s) for each time point, thus keeping the signal at 2,000 Hz. Q_inst_ signal was smoothened by centered moving average over 800 samples (0.04 s) and down-sampled by a factor of 10 to a sampling rate of 2,000 Hz. Only the Q values (together with the corresponding AVA) which where higher than 97% of max{Q_inst_} were selected. This corresponds to the phase of the cycle were the flow is the least pulsatile. From this subset, the averages ($\bar{{\text{Q}}_{\text{peak}}}$, $\bar{{\text{AVA}}_{\text{peak}}}$) were computed for each cardiac output value of each stiffness grade (Figure 3) for further analysis.

### 2.7. Statistics of baseline characteristics

From the experiments with the different valves, the mean and standard deviation of the relative stiffness, mean transvalvular gradient [mmHg], LV work [J], $\bar{{\text{Q}}_{\text{syst}}}$ [ml/s] and $\bar{{\text{Q}}_{\text{syst}}}$ indexed to LVOT [m/s], and maximum AVA [% of LVOT] were reported for each stenosis grade and the 3 following cardiac output values: 0.5, 2.5, and 5.0 l/min in Table 1. In a linear mixed effect model, we tested the fixed effect of the cardiac output and the relative stiffness on each of those 5 variables, setting the valve identifiers as the random effect.

**Table 1:** Baseline characteristics.

**Parameter**	**Cardiac output [l/min]**	**Grade a**	**Grade b**	**Grade c**
Mean transvalvular gradient [mmHg]	0.5	7.5 ± 3.9	11.3 ± 4.1	12.8 ± 4.2
	2.5	12.5 ± 5.2	22.0 ± 5.6	31.7 ± 9.7
	5.0	23.0 ± 7.2	41.9 ± 8.8	61.7 ± 14.3
LV work [J]	0.5	0.15 ± 0.03	0.18 ± 0.04	0.20 ± 0.05
	2.5	0.47 ± 0.06	0.53 ± 0.07	0.61 ± 0.11
	5.0	1.01 ± 0.12	1.20 ± 0.18	1.37 ± 0.21
$\bar{{\text{Q}}_{\text{syst}}}$ [ml/s]	0.5	133 ± 9	135 ± 19	138 ± 22
	2.5	291 ± 15	300 ± 17	307 ± 26
	5.0	508 ± 27	512 ± 26	525 ± 34
$\bar{{\text{Q}}_{\text{syst}}}$ indexed to LVOT [m/s]	0.5	0.24 ± 0.04	0.25 ± 0.07	0.26 ± 0.08
	2.5	0.57 ± 0.08	0.59 ± 0.10	0.60 ± 0.12
	5.0	0.96 ± 0.15	0.96 ± 0.15	0.95 ± 0.18
Maximum AVA [% of LVOT]	0.5	29.0 ± 4.3	12.4 ± 5.7	10.7 ± 6.0
	2.5	30.6 ± 4.6	18.2 ± 5.0	14.3 ± 4.7
	5.0	37.2 ± 6.2	23.1 ± 6.2	18.1 ± 4.1
Relative stiffness value	-	1.00 ± 0.00	1.16 ± 0.08	1.34 ± 0.14

### 2.8. Prediction of relative stiffness and AVA in a modified K-fold cross-validation algorithm

The relative stiffness of each grade was predicted in K-fold cross-validation, a machine learning algorithm (12).

#### 2.8.1. Linear and sigmoid models

First, a linear (with respect to Q) model was used as follows:


(4) $$\begin{array}{l} {\hat{\text{AVA}}}_{1}={\text{F}}_{1}\left(\text{Q},s_{1},\theta_{1},\theta_{2}\right)=\theta_{1}\cdot \text{Q}\cdot \theta_{2}^{s_{1}} \end{array}$$

By analyzing a scatter plot of {Q;AVA} points, we postulated a saturating sigmoid behavior (with respect to Q) and modeled it mathematically as follows:


(5) $$\begin{array}{l} {\hat{\text{AVA}}}_{2}={\text{F}}_{2}(\text{Q},s_{2},\theta_{3},\theta_{4},\theta_{5})=\frac{\theta_{3}}{s_{2}^{\theta_{4}}}(\frac{1}{e^{\left(-\text{Q}\cdot \theta_{5}\right)}+1}-0.5) \end{array}$$

where ${\hat{\text{AVA}}}_{1}$ and ${\hat{\text{AVA}}}_{2}$ are the respective predicted AVA for each model, *s*_1_ and *s*_2_ are the two relative stiffnesses of each model, θ_1,…,5_ are the hyperparameters to be fitted on the training set and to be kept constant for all the valves of the test set and the final clinical decision tool, F_1_ and F_2_ are the two functions describing the relation between those variables.

#### 2.8.2. Fitting of hyperparameters and relative stiffness

**Figure 6:**
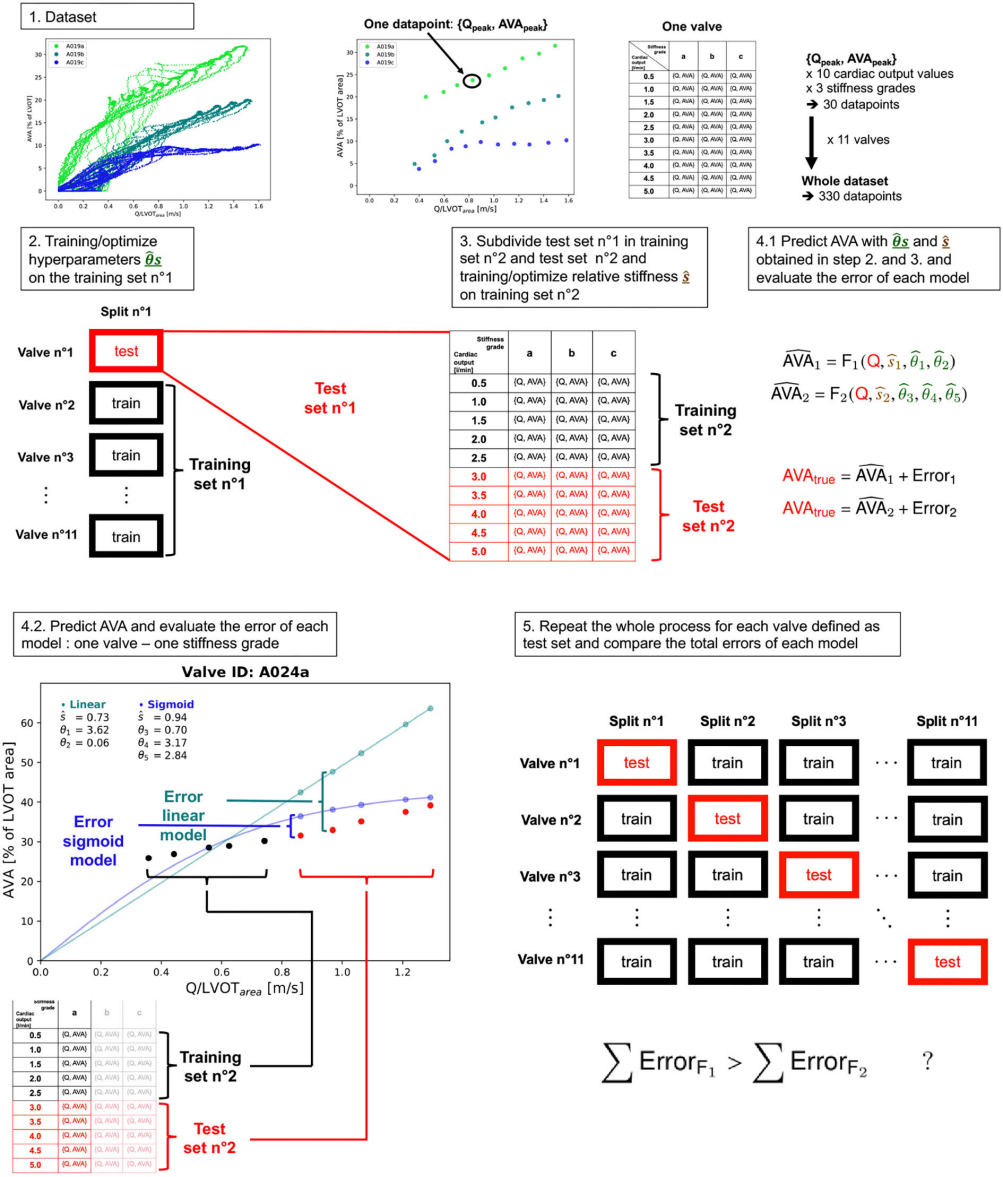
Schematic depiction of the K-fold cross-validation algorithm.

We sequentially trained the hyperparameters θ_1,…,5_ and relative stiffness *s*_1,2_ in a modified K-fold cross-validation algorithm (Figure 6). As previously described (12), we first split the entire dataset comprising $\bar{{\text{Q}}_{\text{peak}}}$ and corresponding AVA and *s* data points of all the analyzed valves into a training dataset which included all the valves except one and a test dataset which included the valve set aside in the training dataset. The entire dataset was composed of *K*=11 valves, with 3 different stiffness grades at 10 different cardiac output values making a total of 330 data points. During the training step (Equation 6), we fitted the parameter θ_1 − 5_ on the training dataset. We repeated the procedure sequentially setting each valve in the test set such that:


(6) $$\begin{array}{l} {\hat{\theta }}^{j}=\underset{\theta }{\text{argmin}}\sum_{i=1}^{m_{\text{1}}}[{\text{AVA}}_{i}-\text{F}({\text{Q}}_{i},s_{i},\theta ){]}^{2} \end{array}$$

where

- *j* ∈ {1,...,*K*} is the index of the split. For each split, there is a training set (noted as $x^{{\text{train}}_{j}}$) and a test set (noted as $x^{{\text{test}}_{j}}$).
- each data point *i* ∈ {1,...,*m*_1_} corresponds to one Q_*i*_ with one AVA_*i*_ at one particular stiffness $s_{i}^{{\text{train}}_{j}}$ of the training set train_*j*_ of size *m*_1_=(*K*−1)·*n*_*s*_·*n*_*f*_. In our case: (*K*−1) = 10 valves, *n*_*s*_ = 3 number stiffness grades and *n*_*f*_ = 10 different cardiac output values. Therefore *m*_1_ = 300.
- ${\hat{\theta }}^{j}$ is the optimal vector ***θ*** ($\theta ={\left[\theta_{1},\theta_{2}\right]}^{⊤}$ for F_1_ and $\theta ={\left[\theta_{3},\theta_{4},\theta_{5}\right]}^{⊤}$ for F_2_) obtained on training set *j*.
- The least square optimization uses the Levenberg-Marquardt algorithm.

Once ${\hat{\theta_{1-5}}}^{j}$ for F_1_ and F_2_ were obtained, we could use them as a fixed variable during the test step (Equation 7) to fit the predicted relative stiffness ŝ^*j*^ on the set composed of the remaining test valve:


(7) $$\begin{array}{l} {\hat{s}}^{j}=\underset{s}{\text{argmin}}\sum_{i=1}^{m_{\text{2}}}[{\text{AVA}}_{i}^{{\text{test}}_{j}}-\text{F}({\text{Q}}_{i}^{{\text{test}}_{j}},s,{\hat{\theta }}^{j}){]}^{2} \end{array}$$

*m*_2_=$n^{{\text{train}}_{j}}\cdot \cdot n_{s}\cdot n_{f}$. In our case: 1 valve, *n*_*s*_=1 stiffness grades *n*_*f*_=10 different cardiac output values, *m*_2_=10. For each test set, the test step was repeated three times for the three stiffness grades.

#### 2.8.3. Accuracy assessment of the linear and sigmoid models

We assessed the accuracy of the models by evaluating the agreement between measured and predicted relative stiffnesses (*s* and ŝ), we computed the Pearson correlation coefficient, the bias, and its respective 95% CIs, the coefficient of variation, and the mean squared error (MSE) by performing a linear regression and Bland-Altman analysis. In order to assess the goodness of fit of the model while taking into account its complexity, we computed the Akaike information criteria (AIC) which we defined as the endpoint (13), with lower values indicating a superior model. The MSE and AIC were computed as follows:


(8) $$\begin{array}{l} \text{MSE}=\frac{1}{n}\overset{n}{\underset{i=1}{\sum }}{\left({\hat{\text{AVA}}}_{1,2}-{\text{AVA}}_{i}\right)}^{2} \end{array}$$

(9) $$\begin{array}{l} \begin{array}{l} \text{AIC}=n\cdot \log\left(\text{MSE}\right)+2k \end{array} \end{array}$$

where *n* is the total number of data points (one for each cardiac output of each stenosis grade of each valve) and *k* is the number of hyperparameters plus one (corresponding to the variance estimate) (14). We reported the mean values of θ_1,…,5_ and their SD obtained during the K-fold cross-validation. Finally, using the same test set, we predicted the $\bar{{\text{AVA}}_{\text{peak}}}$ points corresponding to the $\bar{{\text{Q}}_{\text{peak}}}$ of the 3 and 5 highest cardiac output values, respectively, for each stiffness grade of each valve, by predicting the ŝ_1,2_ using the {$\bar{{\text{Q}}_{\text{peak}}}$, $\bar{{\text{AVA}}_{\text{peak}}}$} data points with *n*_*f*_ = 10-3 = 7 respectively *n*_*f*_ = 10-5 = 5 $\bar{{\text{Q}}_{\text{peak}}}$ of the lowest cardiac output values using Equation (7). We then used ŝ_1,2_ and θ_1,…,5_ and the $\bar{{\text{Q}}_{\text{peak}}}$ of the 3, respectively, 5 highest cardiac output values to predict $\bar{{\text{AVA}}_{\text{peak}}}$ using the linear [Equation (4)] and sigmoid model [Equation (5)]. This prediction scheme takes into account that, in the clinical routine, low-flow low-gradient aortic stenoses are common and require projecting the AVA at normal Q from low Q-values. The number of $\bar{{\text{AVA}}_{\text{peak}}}$ to be predicted (3 and 5) were chosen arbitrarily.

### 2.9. Software used

Data processing and analysis were written in Python and Julia programming languages (15, 16). Image processing was performed in Python. Mixed models were computed using the lme4 packages (17) of R programming language (18).

## 3. Results

**Figure 7:**
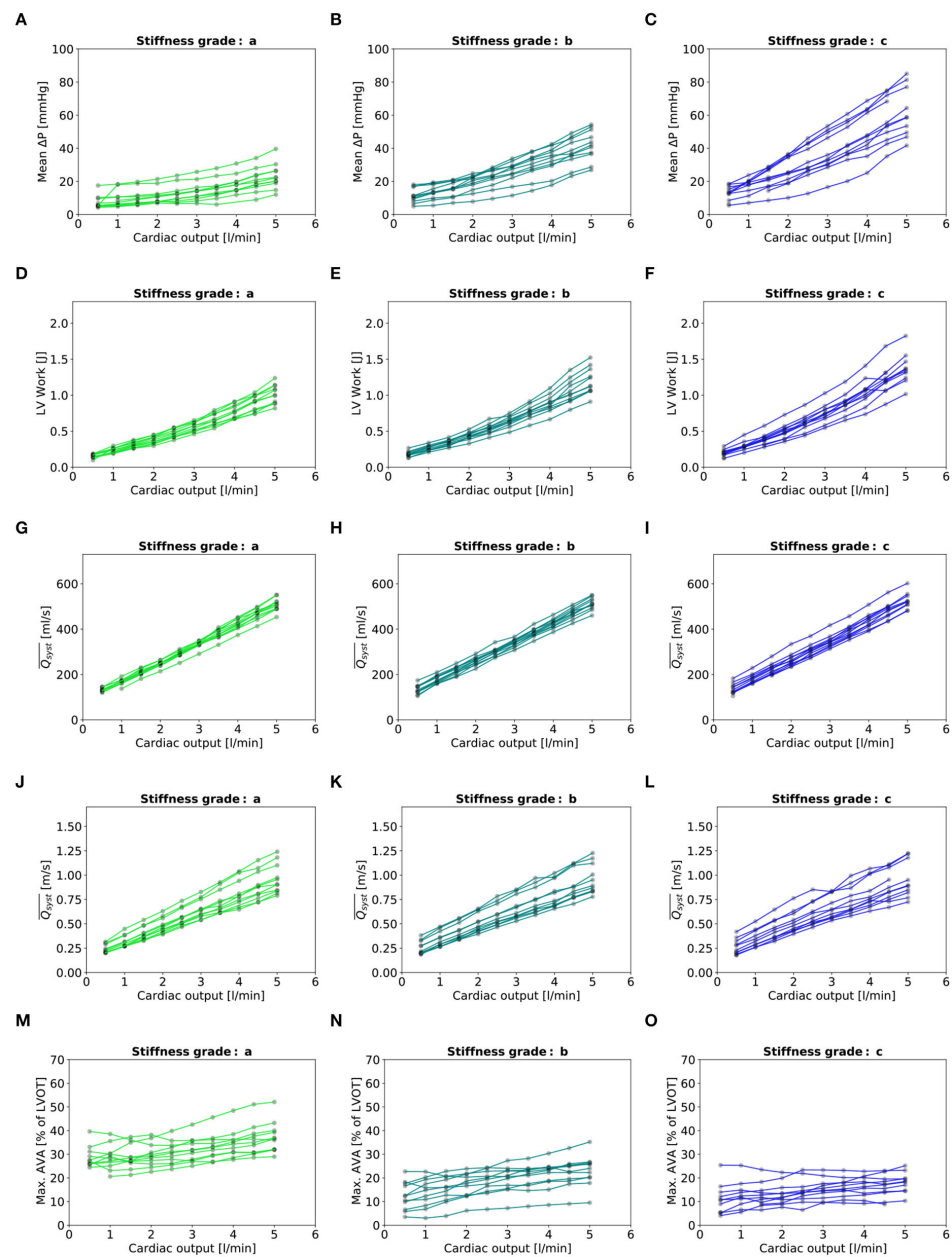
Baseline characteristics: **(A–C)**: Mean transvalvular gradient. **(D–F)**: LV Work. **(G–I)**: $\bar{Q_{\text{syst}}}.\;\text{(J–L)}:\bar{Q_{\text{syst}}}$ indexed to LVOT. **(M–O)**: Maximal AVA. - In each plot, there are eleven lines, each representing one valve.

**Figure 8:**
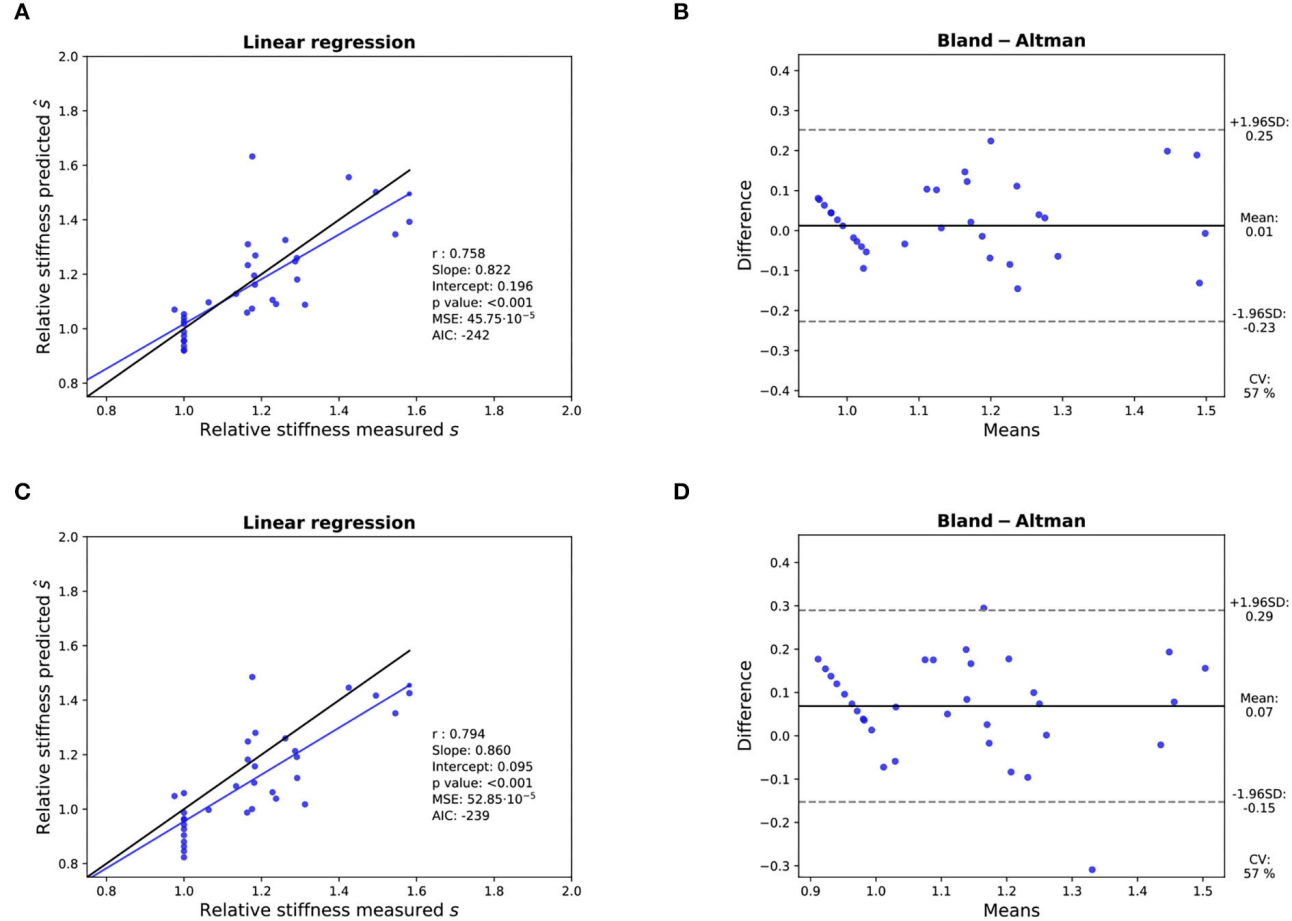
Assessment of the accuracy of the prediction of the relative stiffness - K-fold cross-validation results: **(A,B)**: Linear regression (left) with black line and blue line representing the identity and the linear regression, respectively, and Bland- Altman analysis (right) for 11 valves with the sigmoid model. **(C,D)**: Idem with the linear model. In each linear regression plot, the blue line represents the linear regression between the measured and predicted AVA. The ideal perfect predictions with slope 1 and intercept 0 (the identity) are depicted in black.

**Figure 9:**
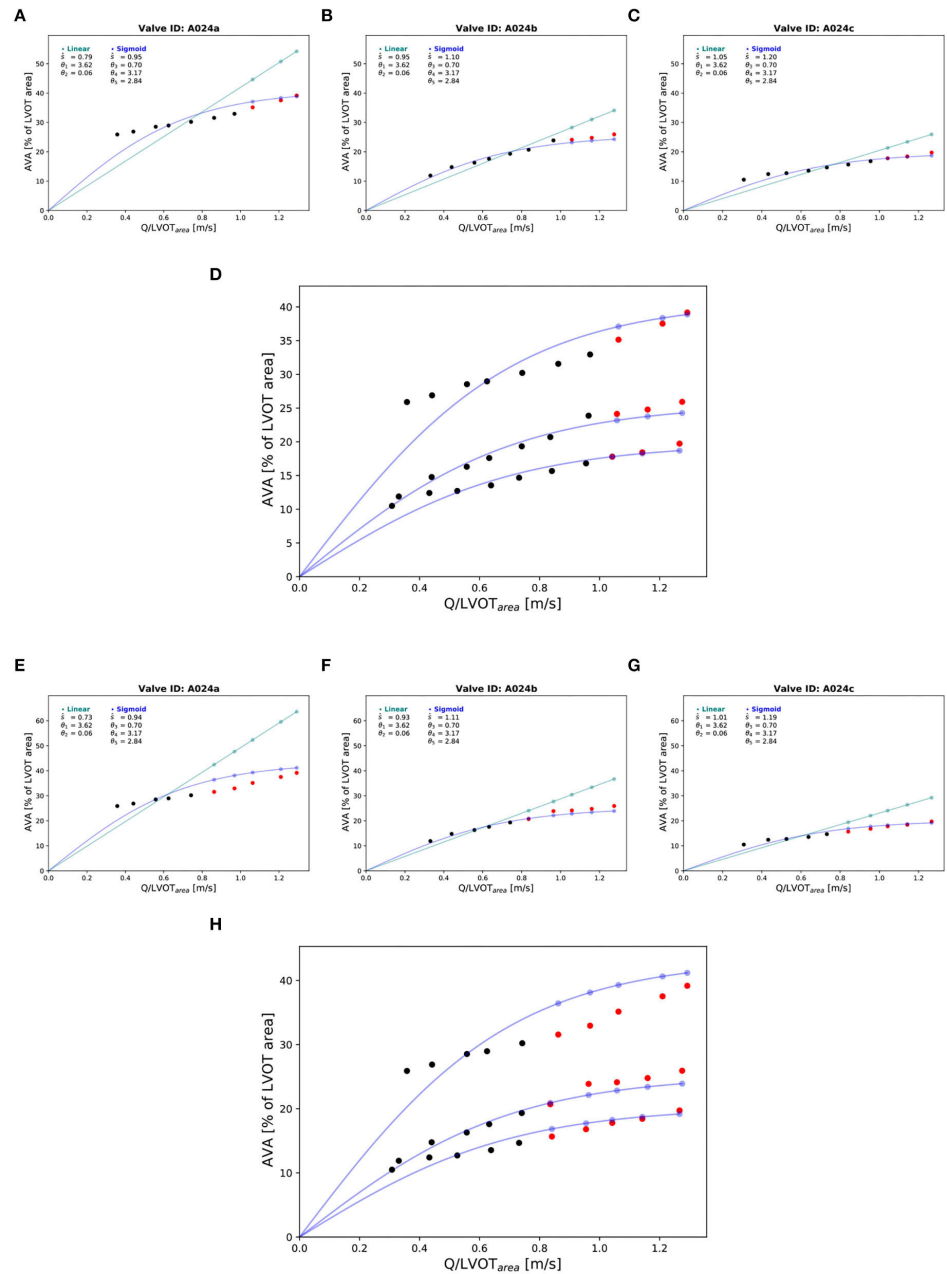
Prediction of the $\bar{AVA_{\text{peak}}}$ of the corresponding $\bar{Q_{\text{peak}}}$ at the highest cardiac output values: “Isostiffness-lines.” One valve (A024) at three different stiffness grades was chosen to illustrate the difference in prediction accuracy between linear (green) and sigmoid (blue) models of the $\bar{AVA_{\text{peak}}}$ corresponding to $\bar{Q_{\text{peak}}}$ with the highest cardiac output values. Prediction of $\bar{AVA_{\text{peak}}}$ at the 3 highest cardiac output values: **(A–C)**. The black points are true AVA used for the prediction. The red points are the true AVA set aside (not used for prediction). The points in color are the predicted AVA with their corresponding lines for the two respective models. Combination of the three stiffness grades data points and corresponding prediction of three “isostiffness”- lines of the sigmoid model: **(D)**. Prediction of AVA at the 5 highest cardiac output values: **(E–G)**. Combination of the three stiffness grades data points and corresponding prediction of three “isostiffness” lines of the sigmoid model: **(H)**.

**Figure 10:**
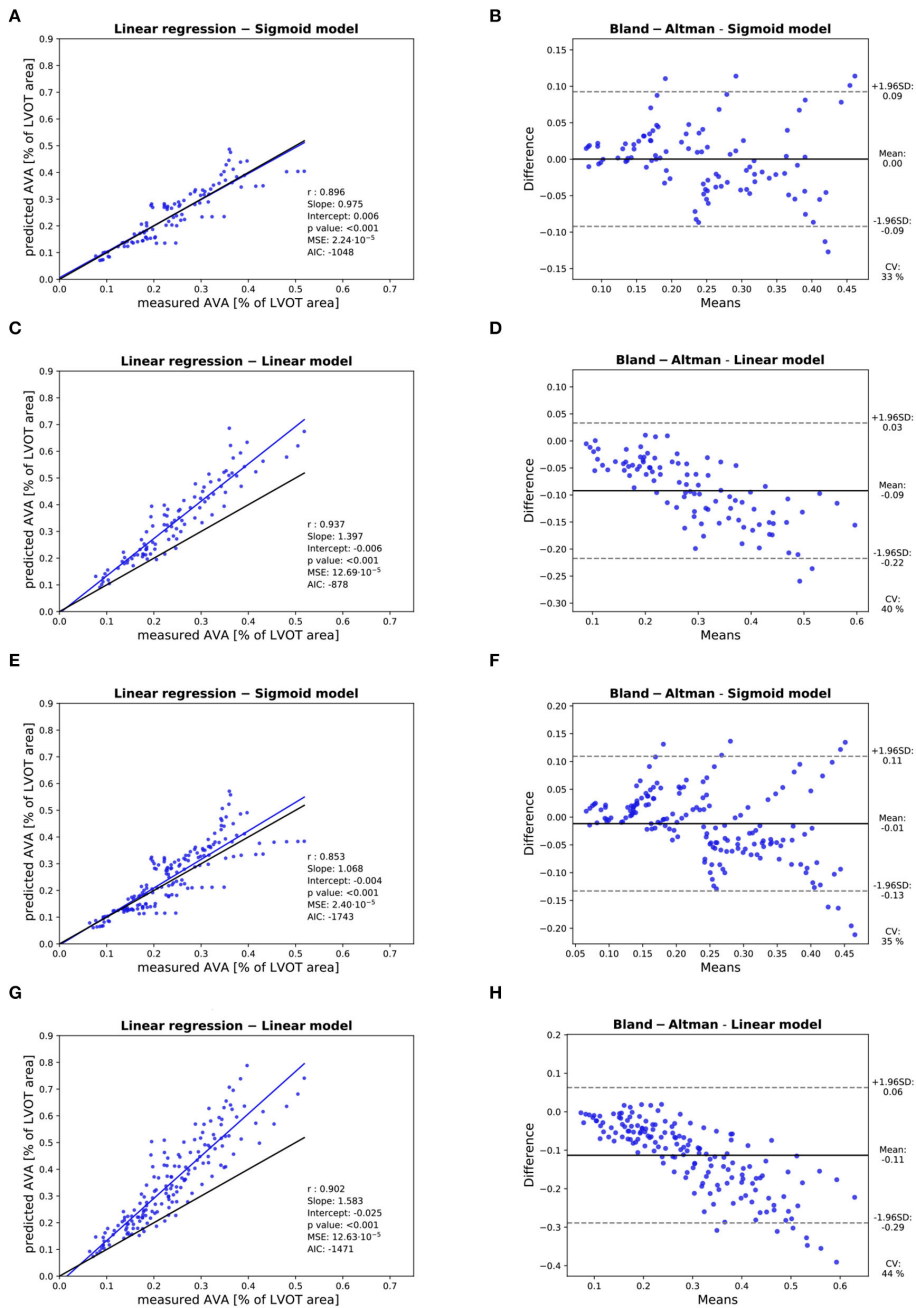
Assessment of the accuracy of the prediction of the $\bar{AVA_{\text{peak}}}$: Assessment of the $\bar{AVA_{\text{peak}}}$ predicted with the linear model with $\bar{Q_{\text{peak}}}$ of the 3 highest cardiac output values with linear regression **(A)** and Bland-Altman analysis **(B)**. Assessment of the $\bar{AVA_{\text{peak}}}$ predicted with the sigmoid model with $\bar{Q_{\text{peak}}}$ of the 3 highest cardiac output values with linear regression **(C)** and Bland-Altman analysis **(D)**. Assessment of the $\bar{AVA_{\text{peak}}}$ predicted with the linear model with $\bar{Q_{\text{peak}}}$ of the 5 highest cardiac output values with linear regression **(E)** and Bland-Altman analysis **(F)**. Assessment of the $\bar{AVA_{\text{peak}}}$ predicted with the sigmoid model with $\bar{Q_{\text{peak}}}$ of the 5 highest cardiac output values with linear regression **(G)** and Bland-Altman analysis **(H)**. In each linear regression plot, the blue line represents the linear regression between the measured and predicted $\bar{AVA_{\text{peak}}}$. The ideal perfect predictions with slope 1 and intercept 0 (the identity) are depicted in black.

**Figure 11:**
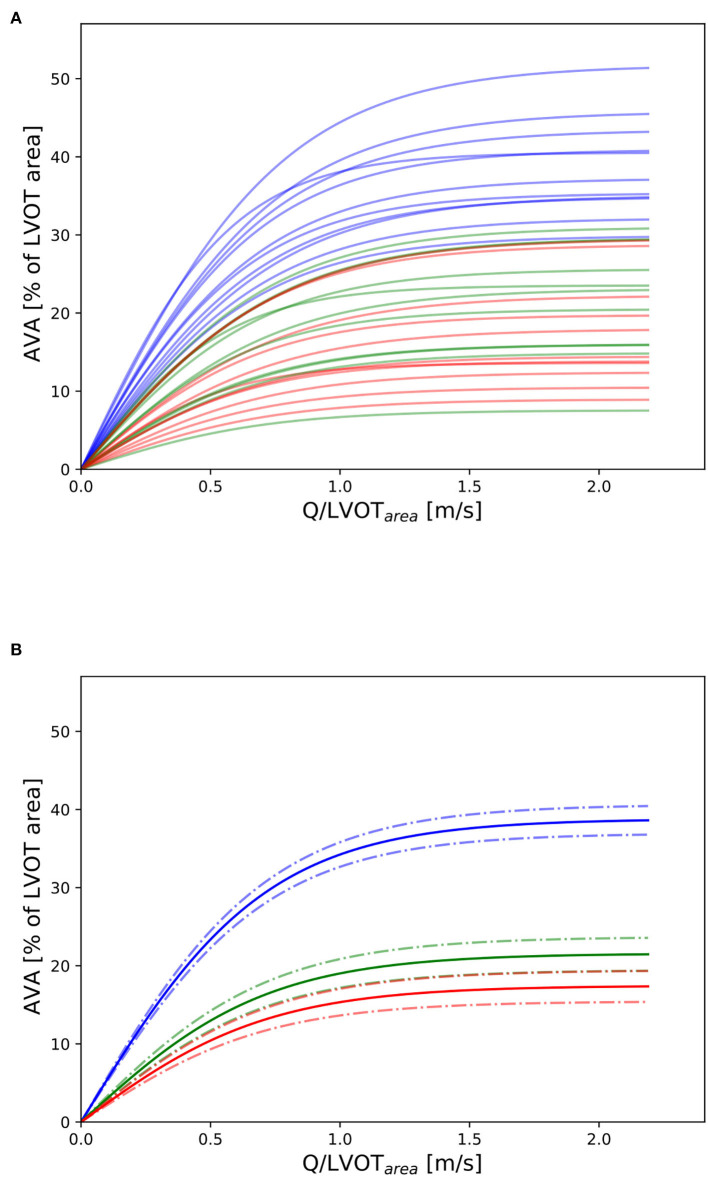
Nomogram: **(A)** Depiction of all “isostiffness-lines” of all the valves with grades 0, 1, and 2 in blue, green, and red, respectively. **(B)** Mean and corresponding confidence interval (± standard error) of the “isostiffness-line” for each stiffness grade. A similar theoretical nomogram with different “isostiffness-lines” could be used in clinical practice to classify aortic stenosis severity, for any valve size, at any flow rate.

Three valves were excluded from the data analysis because their neoprene sheet was accidentally torn during the valve preparation process. The baseline characteristics of the 11 valves included in the final data analysis are presented in the Figure 7 and Table 1. There were 4 data points missing making a total of 330-4=326 effective data points {$\bar{{\text{Q}}_{\text{peak}}}$, $\bar{{\text{AVA}}_{\text{peak}}}$}. The obtained $\bar{{\text{Q}}_{\text{syst}}}$ largely encompassed the reported mean physiological Q encountered in the clinic (134 ± 8 to 508 ± 28 ml/s at 0.5 and 5.0 l/min read at the flow probe). At normal physiological flow (cardiac output of 5.0 l/min) and native stiffness grade, there was a $\bar{{\text{Q}}_{\text{syst}}}$ of 508 ± 27 ml/s, $\bar{{\text{Q}}_{\text{syst}}}$ indexed to LVOT of 0.96 ± 0.15 m/s, a maximum AVA of 37.2 ± 6.5 % of LVOT, a transvalvular mean gradient of 23.3 ± 7.5 mmHg and a W_max_ of 1.01 ± 1.01 J. There was a significant positive effect of the cardiac output on those five 5 variables: (*p* < 0.001, Table 2). On the other hand, both the $\bar{{\text{Q}}_{\text{syst}}}$ (*p* = 0.277) and the $\bar{{\text{Q}}_{\text{syst}}}$ indexed to LVOT (*p* = 0.378) were not influenced by the relative stiffness, confirming that $\bar{{\text{Q}}_{\text{syst}}}$ was, as expected, very similar between different stiffness grades. Moreover, the relative stiffness had a significant negative effect on the maximum AVA and a significant positive effect on both the LV work and the mean transvalvular gradient (p<0.001 for the three values, Table 2). The linear model F_1_ could predict the stiffness with good accuracy (ŝ_1_=0.860 · *s*_1_ + 0.095, *r* = 0.794, *p* < 0.001, θ_1_=3.69 ± 0.66, θ_2_ = 0.066 ± 0.009) with a higher bias and equally high coefficient of variation compared to the sigmoid model (bias: 0.07, 95% CI = [–0.15; 0.29], CV: 57%, Figures 8C,D). The sigmoid model F_2_ could predict the relative stiffness with good accuracy (ŝ_2_= 0.822 · *s*_2_ + 0.196, *r* = 0.758, *p* < 0.001, θ_3_=0.72 ± 0.01, θ_4_=3.14 ± 0.11, θ_5_=2.80 ± 0.23) with a relatively low bias but a high coefficient of variation (bias: 0.01, 95% CI = [–0.23; 0.25], CV = 57%, Figures 8A,B). Overall, the sigmoid model better predicted the relative stiffness than the linear model (AIC: –242 vs. –239). The sigmoid models also better predicted the $\bar{{\text{AVA}}_{\text{peak}}}$ corresponding to the $\bar{{\text{Q}}_{\text{peak}}}$ of the 3 (AIC = –1,743 vs. AIC = –1,048) and 5 highest cardiac output values (AIC = –1,471 vs. AIC = -878) than the linear model for each stiffness grade of each valve (Figure 9). The MSE was more than five times higher in the linear model than in the sigmoid model (MSE = 12.69e^−5^ vs. 2.24e^−5^ and MSE = 12.63e^−5^ vs. 2.40e^−5^ for the prediction of $\bar{{\text{AVA}}_{\text{peak}}}$ with $\bar{{\text{Q}}_{\text{peak}}}$ of the 3 and 5 highest cardiac output values, respectively). The linear model systematically overestimated the predicted $\bar{{\text{AVA}}_{\text{peak}}}$ as can be observed with the slope value (slope = 1.40 respectively slope = 1.58 for 3 respectively, 5 $\bar{{\text{AVA}}_{\text{peak}}}$ predictions) whereas there was no such bias in the sigmoid model in which the slope was much closer to 1 (slope = 0.98 respectively slope = 1.07 for 3 respectively, 5 AVAs predictions (Figure 10). Interestingly, even after having carefully subtracted the delay between the signal allowing to synchronize the Q_inst_ and the AVA_inst_ signal, we observed that a subset of points had positive computed Q_inst_ with a closed valve. This could be attributed to a bulging effect of the valve where the cusps move during the isovolumetric contraction time without opening (Figure 3C). Finally, we plotted all the “isostiffness-lines” of all the valves on a single plot (Figure 11A). Due to the cross-validation, every valve has different θ which explains why some lines cross each other (which would not be the case with unified θ. Moreover, the relative stiffness of the valve A028*b* had the highest relative stiffness and was higher than the relative stiffness of A028*c* (Figure 11A). We plotted the corresponding mean value of the “isostiffness-lines” of each group with their corresponding confidence interval (Figure 11B).

**Table 2:** Effect of cardiac output and stiffness grade.

**Effects**	**Parameters**	**Mean**	**95% CI**	***p* val**
Effect of cardiac output	Maximum AVA [% of LVOT]	2.0	[1.3;2.6]	<0.001
	$\bar{{\text{Q}}_{\text{syst}}}$ [ml/s]	84.0	[83.0;85.0]	<0.001
	$\bar{{\text{Q}}_{\text{syst}}}$ indexed to LVOT [m/s]	0.16	[0.15;0.16]	<0.001
	LV work [J]	0.22	[0.21;0.23]	<0.001
	Mean transvalvular gradient [mmHg]	7.0	[6.1;7.9]	<0.001
Effect of stiffness grade	Maximum AVA [% of LVOT]	−40.0	[−44.2;−35.9]	<0.001
	$\bar{{\text{Q}}_{\text{syst}}}$ [ml/s]	45.0	[−36.0;126.0]	0.277
	$\bar{{\text{Q}}_{\text{syst}}}$ indexed to LVOT [m/s]	0.07	[−0.09;0.24]	0.378
	LV work [J]	0.49	[0.26;0.72]	<0.001
	Mean transvalvular gradient [mmHg]	57.1	[48.5;65.7]	<0.001

## 4. Discussion

In this *in vitro* experiment, we could successfully implant harvested porcine valves in a flow loop simulating the left heart with physiological afterload. We could impose a broad spectrum of cardiac output and corresponding Q encompassing both physiological normal-flow and low-flow encountered in real patients being referred for evaluation for aortic valve replacement in case of severe aortic stenosis. We could reliably stiffen the valves chemically to obtain an *in vitro* model of aortic stenosis. The calculated LV work was well aligned with the work per beat reported in the literature (19). Most importantly, the sigmoid model predicted more accurately the $\bar{{\text{AVA}}_{\text{peak}}}$ at high $\bar{{\text{Q}}_{\text{peak}}}$, a challenging and frequent situation for clinicians performing low-dose dobutamine stress echocardiography in patients with aortic stenosis. The non-linear behavior of hemodynamic parameters such as transvalvular pressure loss and peak flow rate $\bar{{\text{Q}}_{\text{peak}}}$ have previously been described in past (8). In order to be usable in the clinic, the model had to be simple and should take parameters that can be easily measured in echocardiography such as AVA, Q, and the area of the LVOT. The modified sigmoid function presented in this manuscript which meets those two design constraints has been found empirically. This study gives hope of constructing a nomogram with “isostiffness-lines” over the entire clinically relevant spectrum of Q- and AVA-values. With such a nomogram, the intrinsic stiffness of individual aortic valves could be defined independently of the instantaneous Q at the time of patient evaluation. Clinical scenarios include any form of cardiac decompensation at the advanced stage of aortic stenosis when Q is low. A unique iso-stiffness value must, therefore, be determined to define the limit between severe and non-severe aortic stenosis. This value could then be used as a threshold to refer the patient to aortic valve replacement without the need of performing additional tests to increase Q, such as dobutamine stress echocardiography. This will require validation in the clinics but has the potential to simplify the evaluation of patients with aortic stenosis.

## 5. Limitations

We could not subtract the retrograde flow of valve A017 because the flow sensor was positioned proximal to the left atrium. However, we decided to include the valve in the final analysis as the amplitude of the retrograde flow at the phase of the ejection time selected for the cross-validation was small as seen in Figure 3C. We observed that valve number A028 had a slightly higher AVA at grade *c* than at grade *b*. Although this underlines the imperfection of the stiffening procedure, we included this valve in the analysis as the corresponding computed relative stiffness at grade *c* was also lower than at grade *b* and the algorithm did not make any assumptions about the order of the stiffness grade. Due to the physical limitations of the compliance chamber, the range of diastolic pressure varied at extreme (both low and high) cardiac output values (Figures 4B,C). As the pressure was measured in the compliance chamber at 20.5 cm downstream of the valve annulus, full pressure recovery was allowed (20) and effects of turbulent flow immediately downstream of the valve orifice were avoided. The transvalvular pressure loss was slightly overestimated due to the viscous losses in the ascending aorta (15.5 cm). This additional loss was estimated according to Poiseuille's law at approximately 0.01 mmHg, which seems acceptably small (although the actual loss was probably somewhat higher due to pulsatility). Formaldehyde stiffens the valve tissue by protein cross-linking but does not reflect the calcification process of the valves. It also assumes a uniform stiffening and not a focal stiffening of the tip of the cusps which can be encountered in the clinic. We attempted other stiffening procedures by applying tar to the valve which resulted in the unsatisfying loss of integrity of the valve. The AVA with respect to LVOT was small. We attribute this effect to our suturing technique which could not completely place the valve under the same dynamic tensile conditions as seen during physiological systole. This also explains the relatively high transvalvular gradient obtained at physiological Q. As we think that it is physically difficult to conceive an infinite AVA with infinite Q, a saturating effect was a prerequisite for a model candidate corresponding to this physical constraint. The sigmoid functions have such a characteristic. Although we could prove the higher accuracy of the sigmoid model over the linear model, we acknowledge that this sigmoid model does not capture most of the other complex physical phenomena involved in the process of the opening of a valve under the constraint of transvalvular flow. Therefore, a function better describing this relation probably exists.

## Data availability statement

The raw data supporting the conclusions of this article will be made available by the authors, upon reasonable request.

## Ethics statement

Review and approval by the ethics committee was not required for this study.

## Author contributions

EB: study design, data acquisition, creation of figures, data analysis, and writing of manuscript. MS: data acquisition and data analysis. SZ: study design and data analysis. MC: creation of figures. DO and SM: study design and writing of manuscript. CS: writing of manuscript. All authors contributed to the article and approved the submitted version.

## Funding

This work has been supported by the Bern Center for Precision Medicine [Canton of Bern, University of Bern and the University Hospital of Bern (Inselspital), Switzerland] and the Gottfried und Julia Bangerter-Rhyner Foundation (Basel, Switzerland).

## Conflict of interest

The authors declare that the research was conducted in the absence of any commercial or financial relationships that could be construed as a potential conflict of interest.

## Publisher's note

All claims expressed in this article are solely those of the authors and do not necessarily represent those of their affiliated organizations, or those of the publisher, the editors and the reviewers. Any product that may be evaluated in this article, or claim that may be made by its manufacturer, is not guaranteed or endorsed by the publisher.
